# Supplementary figures and images for: Mathematical modeling to understand the role of bivalent thrombin-fibrin binding during polymerization
Source: PLoS Comput Biol. 2022 Sep 15;18(9):e1010414. doi: 10.1371/journal.pcbi.1010414 (PMC9477365; doi:10.1371/journal.pcbi.1010414)

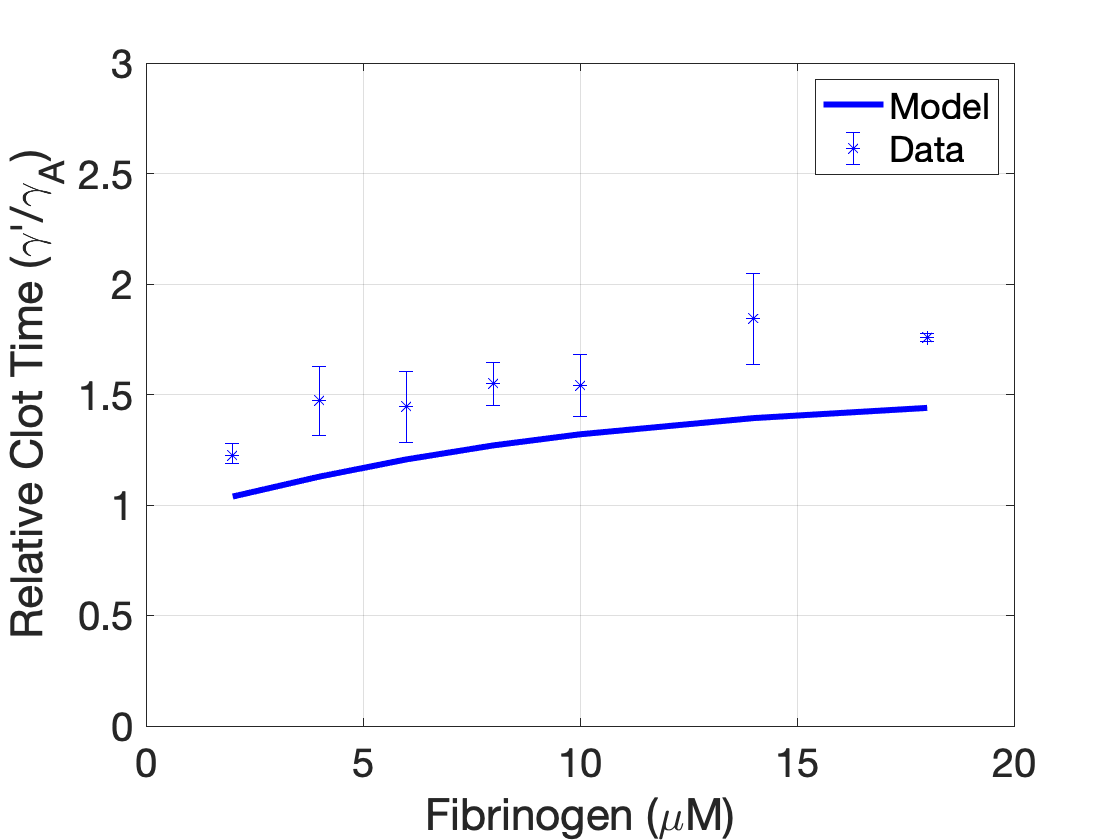

Supplement: S1 Fig — The ratios of the relative clot times between γA/γ′ to γA/γA fibrinogen, with clot time being defined as the time to half-maximal turbidity. The model output (solid line) shows a similar trend but falls slightly below the experimental data from Kim et al. [38] (dots with error bars). To simulate the effects of Batroxobin, FpB cleavage was turned off and the dissociation constant for thrombin with the E-domain was decreased according to rates from the literature [45]. There was a marked increase in protofibril number per fiber, an increased lag time, and an overall decrease in protofibril number per fiber when comparing γA/γ′ to γA/γA. (TIF) [file pcbi.1010414.s001.tif]
