# Supplementary material for: Mathematical modeling to understand the role of bivalent thrombin-fibrin binding during polymerization
Source: PLoS Comput Biol. 2022 Sep 15;18(9):e1010414. doi: 10.1371/journal.pcbi.1010414 (PMC9477365; doi:10.1371/journal.pcbi.1010414)
Supplement: S1 Appendix — (PDF) [file pcbi.1010414.s002.pdf]

S1 Appendix: Mathematical equations describing the full fibrin polymerization model

$$\frac{d[f_{ab}]}{dt} = -\,k_a^+ T[f_{ab}] + k_a^-[Ef_{ab}] \tag{1}$$

$$\frac{d[Ef_{ab}]}{dt} = +\,k_a^+ T[f_{ab}] - k_a^-[Ef_{ab}] - k_{cat,a}[Ef_{ab}] \tag{2}$$

$$\begin{aligned}\frac{d[f_b]}{dt} = &-\,k_b^+ T[f_b] + k_a^-[Ef_b] + k_{cat,a}[Ef_{ab}] \\ &-\,k_{pi}[f_b]\left(2[f_b] + [f] + [f_2] + [f_3] + \cdots + [f_{10}]\right) \\ &-\,k_{pg}[f_b][f_n]\end{aligned} \tag{3}$$

$$\frac{d[Ef_b]}{dt} = +\,k_b^+ T[f_b] - k_a^-[Ef_b] - \alpha k_{cat,b}[Ef_b] \tag{4}$$

$$\begin{aligned}\frac{d[f]}{dt} = &+\,\alpha k_{cat,b}[Ef_b] \\ &-\,k_{pi}[f]\left(2[f] + [f_b] + [f_2] + [f_3] + \cdots + [f_{10}]\right) \\ &-\,k_{pg}[f][f_n]\end{aligned} \tag{5}$$

$$\begin{aligned}\frac{d[f_2]}{dt} = &+\,k_{pi}([f]^2 + [f_b]^2 + [f][f_b]) \\ &-\,k_{pi}[f_2]\sum_{j=1}^{10}[f_j] - k_{pi}[f_2]([f] + [f_b]) \\ &-\,k_{pg}[f_2][f_n]\end{aligned} \tag{6}$$

$$\begin{aligned}\frac{d[f_i]}{dt} = &+\,k_{pi}[f_{i-1}]\left([f] + [f_b]\right) + k_{pi}\sum_{j+k=i}^{2\le j\le k}[f_j][f_k] \\ &-\,k_{pi}[f_i]\left([f] + [f_b]\right) - k_{pi}[f_i]\sum_{j=1}^{10}[f_j] \\ &-\,k_{pg}[f_i][f_n]\end{aligned} \tag{7}$$

$$\begin{aligned}\frac{d[f_n]}{dt} = &+\,k_{pi}[f_{10}]\left([f] + [f_b]\right) + k_{pi}\sum_{j+k\ge 11}^{2\le j\le k\le 10}[f_j][f_k] \\ &-\,2k_{fi}[f_n^p]^2 - k_{fg}[f_n^p][f_r]\end{aligned} \tag{8}$$

$$[f_n^p] = \phi_{pol}\cdot[f_n] \tag{9}$$

$$\frac{d[f_r]}{dt} = +k_{fi}[f_n^p]^2 \tag{10}$$

$$\frac{d[f^n_{tot}]}{dt} = +\,2k_{fi}[f^p_n]^2 + k_{fg}[f^p_n][f_r] \tag{11}$$

$$f(BE_1, BE, BG) = k_{b,cat}(S_\alpha BE_1 + S_\beta BE + S_\gamma B) \tag{12}$$

$$\begin{aligned} \frac{d[C_b]}{dt} =& 2k_{pi}[f_b]^2 + k_{pi}[f_b][f] \\ &+ k_{pi}\sum_{j=2}^{9}[f_b][f_j] \\ &- 10k_{pi}(\phi_b)[f_{10}]([f] + [f_b]) \\ &- 11k_{pi}(\phi_b)\left(\sum_{j+k=11}^{2\le j\le k}[f_j][f_k]\right) \\ &- k_{pi}(\phi_b)\left(\sum_{j+k\ge 11}^{2\le j\le k\le 10}[f_j][f_k](j+k)\right) \\ &- f(BE_1, BE, BG)(\phi_{cb}) \end{aligned} \tag{13}$$

$$\begin{aligned} \frac{d[C_f]}{dt} =& 2k_{pi}[f]^2 + k_{pi}[f_b][f] \\ &+ k_{pi}\sum_{j=2}^{9}[f][f_j] \\ &- 10k_{pi}(1-\phi_b)[f_{10}]([f] + [f_b]) \\ &- 11k_{pi}(1-\phi_b)\left(\sum_{j+k=11}^{2\le j\le k}[f_j][f_k]\right) \\ &- k_{pi}(1-\phi_b)\left(\sum_{j+k\ge 11}^{2\le j\le k\le 10}[f_j][f_k](j+k)\right) \\ &+ f(BE_1, BE, BG)(\phi_{cb}) \end{aligned} \tag{14}$$

$$
\begin{aligned}
\frac{d[C_{fb}]}{dt} =& k_{pi}[f_b][f_{10}] + 10k_{pi}(\phi_b)[f_{10}]([f] + [f_b]) \\
& + 11k_{pi}(\phi_b)\left(\sum_{j+k=11}^{2\le j\le k}[f_j][f_k]\right) \\
& + k_{pg}[f_n][f_b] \\
& + k_{pi}(\phi_b)\left(\sum_{j+k\ge 11}^{2\le j\le k\le 10}[f_j][f_k](j+k)\right) \\
& S_{agg}(-k_{fi}[f_n^p][C_{fb}] - k_{fg}[f_r][C_{fb}]) \\
& + k_{pg}(\phi_b)\sum_{j=1}^{10}[f_j][f_n](j) \\
& - f(BE_1, BE, BG)(\phi_{C_{fb}})
\end{aligned} \tag{15}
$$

$$
\begin{aligned}
\frac{d[C_{fn}]}{dt} =& k_{pi}[f][f_{10}] + 10k_{pi}(1-\phi_b)[f_{10}]([f] + [f_b]) \\
& + 11k_{pi}(1-\phi_b)\left(\sum_{j+k=11}^{2\le j\le k}[f_j][f_k]\right) \\
& + k_{pi}(1-\phi_b)\left(\sum_{j+k\ge 11}^{2\le j\le k\le 10}[f_j][f_k](j+k)\right) \\
& + k_{pg}[f_n][f] \\
& - k_{fi}[f_n^p][C_{fn}] - k_{fg}[f_r][C_{fn}] \\
& + k_{pg}(1-\phi_b)\sum_{j=1}^{10}[f_j][f_n](j) \\
& + f(BE_1, BE, BG)(\phi_{C_{fb}})
\end{aligned} \tag{16}
$$

$$
\begin{aligned}
\frac{d[C_{fr}]}{dt} =& + 2k_{fi}[f_n^p][C_{fn}] + k_{fg}[C_{fn}][f_r] \\
& S_{agg}(+k_{fi}[f_n^p][C_{fb}] + k_{fg}[f_r][C_{fb}])
\end{aligned} \tag{17}
$$

$$
\begin{aligned}
\frac{dT}{dt} =& - k_{on,E}[T][E_1] + k_{off,E}[BE_1] \\
& - k_{on,E}[T][E] + k_{off,L}[BE] \\
& - k_{on,G}[T][G_1] + k_{off,G}[BG_1] \\
& - k_{on,G}[T][G] + k_{off,G}[B_G] \\
& - k_a^+T[f_{ab}] + k_a^-[Ef_{ab}] + k_{cat,a}[Ef_{ab}] \\
& - k_b^+T[f_b] + k_b^-[Ef_b] + k_{cat,b}[Ef_b] \\
& + k_{b,cat}(\phi_{cb} + \phi_{C_{fb}})\,(S_\alpha BE_1 + S_\beta BE + R_{biv}S_\gamma B)
\end{aligned} \tag{18}
$$

$$\frac{d[E_1]}{dt} = -k_{on,E}[T][E_1] + k_{off,E}[BE_1] + dE1 + k_{b,cat}S_\alpha[BE_1](\phi_{cb} + \phi_{C_{fb}}) \tag{19}$$

$$\frac{d[BE_1]}{dt} = +k_{on,E}[T][E_1] - k_{off,E}[BE_1] + dBE1 - k_{b,cat}s_\alpha[BE_1](\phi_{cb} + \phi_{C_{fb}}) \tag{20}$$

$$\begin{aligned}\frac{d[E]}{dt} =& -k_{on,E}[T][E] + k_{off,L}[BE] \\ & -k_{on,E2}[B_G] + k_{off,E2}[B] + dE - k_{b,cat}S_\beta[BE](\phi_{cb} + \phi_{C_{fb}})\end{aligned} \tag{21}$$

$$\begin{aligned}\frac{d[B_E]}{dt} =& +k_{on,E}[T][E] - k_{off,E}[B_E] \\ & -k_{on,G2}[B_E] + k_{off,E}[B] + dBE + k_{b,cat}S_\beta[BE](\phi_{cb} + \phi_{C_{fb}})\end{aligned} \tag{22}$$

$$\frac{dG_1}{dt} = -k_{on,G}[T][G_1] + k_{off,G}[BG_1] + dG1 \tag{23}$$

$$\frac{dBG_1}{dt} = +k_{on,G}[T][G_1] - k_{off,G}[BG_1] + dBG1 \tag{24}$$

$$\begin{aligned}\frac{dG}{dt} =& -k_{on,G}[T][G] + k_{off,G}[B_G] \\ & -k_{on,G2}[B_{L'}] + k_{off,G}[B] + dG + k_{b,cat}S_\gamma[B](\phi_{cb} + \phi_{C_{fb}})(R_{biv})\end{aligned} \tag{25}$$

$$\begin{aligned}\frac{dB_G}{dt} =& +k_{on,G}[T][G] - k_{off,G}[B_G] \\ & -k_{on,L2}[B_G] + k_{off,L}[B] + dBG - k_{b,cat}S_\gamma[B](\phi_{cb} + \phi_{C_{fb}})(1 - R_{biv})\end{aligned} \tag{26}$$

$$\begin{aligned}\frac{dB}{dt} =& +k_{on,G2}[B_{L'}] - k_{off,G}[B] \\ & +k_{on,L2}[B_G] - k_{off,L}[B] - k_{b,cat}S_\gamma[B](\phi_{cb} + \phi_{C_{fb}})(1 - R_{biv})\end{aligned} \tag{27}$$

$$\begin{aligned}dfbp =& -k_{pi}[f_b]\left(2[f_b] + [f] + [f_2] + [f_3] + \cdots + [f_{10}]\right) \\ & -k_{pg}[f_b][f_n]\end{aligned} \tag{28}$$

$$\begin{aligned}dfp =& -k_{pi}[f]\left(2[f] + [f_b] + [f_2] + [f_3] + \cdots + [f_{10}]\right) \\ & -k_{pg}[f][f_n]\end{aligned} \tag{29}$$

$$\begin{aligned}dE1 =& +2 * k_{bcat}[Efb] \\ & +dfbp(2 - \phi_{gp}) \\ & -dfp(\phi_{gp})(1 - \phi_{BE1})\end{aligned} \tag{30}$$

$$dBE1 = -dfp(\phi_{gp})(\phi_{BE1}) \tag{31}$$

$$\begin{aligned}dE =& +dfbp(\phi_{gp}) \\ & +dfp(2 - \phi_{gp})(1 - \phi_{BE1})\end{aligned} \tag{32}$$

$$dBE = +dfp(\phi_{gp})(\phi_{BE1}) \tag{33}$$

$$dG1 = (-dfp(\phi_{gp}) - dfbp(\phi_{gp}))(1 - \phi_{BG1}) \tag{34}$$

$$dBG1 = (-dfp(\phi_{gp}) - dfbp(\phi_{gp}))(\phi_{BG1}) \tag{35}$$

$$dG = (-dfp(\phi_{gp}) - dfbp(\phi_{gp}))(1 - \phi_{BG1}) \tag{36}$$

$$dBG = (-dfp(\phi_{gp}) - dfbp(\phi_{gp}))(\phi_{BG1}) \tag{37}$$
