# Supplementary material for: Mathematical modeling to understand the role of bivalent thrombin-fibrin binding during polymerization
Source: PLoS Comput Biol. 2022 Sep 15;18(9):e1010414. doi: 10.1371/journal.pcbi.1010414 (PMC9477365; doi:10.1371/journal.pcbi.1010414)
Supplement: Attachment — Submitted filename: ResposeToReviewers_7422.pdf [file pcbi.1010414.s003.pdf]

“Mathematical modeling to understand the role of bivalent thrombin binding during fibrin polymerization” by Michael Kelley and Karin Leiderman

Response to Reviewers: 7/1/2022

First, we thank the editors and reviewers for taking the time to review our paper. The comments were helpful and we believe improved the clarity of our study. Below we address the comments from reviewers. We hope that the revised work meets the standards for publication. Our responses are in blue.

Reviewer #1: The manuscript titled "Mathematical modeling to understand the role of bivalent thrombin-fibrin binding during
polymerization" presents a new method to simulate the fibrin polymerization that considers fibrin, fibrinogen and thrombin.
I have some minor concerns on the paper as below.

Comments:

1) The manuscript is nicely written and well explained.

We thank the reviewer for the kind words.

2) A flowchart showing various methods and steps would have been of interest for the layman readers. This will help to understand the link between the SBM, polymerization and final results.

We agree that there were a lot of moving parts in the paper and appreciate the change to clarify. We have added a flowchart as a new figure the paper.

3) Despite of the tremendous effort that the authors put forward in this paper, the outcome of the model is not so appealing. Especially, when validating/comparing the results with the experiments, the comparison is made about protofibril number, distance between protofibrils through thrombin concentration and fibrinogen type. This means the spacio-temporal feature of polymerization process is absent, which is disappointing.

It is unfortunate that the reviewer was disappointed in the outcome of the paper. We agree that this model would also be useful to use in a spatial-temporal context and that is definitely work to do in the near future. But, the main goal of this paper was to first gain insight into the roles of gamma’ fibrinogen during polymerization. We think that the hypothesis about a dual role for gamma’ fibrinogen is novel and interesting since it seems to be mostly thought of as a sink for thrombin in the current literature. Future work will include embedding aspects of this model into spatial-temporal models previously published from our group (Leiderman & Fogelson 2010 MMB, and 2012 BMB). We have also added text in the discussion related to this and further comments

by reviewer 2 that speak to this issue. Please see that comment below in the responses to reviewer 2.

Reviewer #2: This is a rigorous, detailed computational study of fibrin-thrombin interactions during polymerization, with a focus on the role of gamma’ fibrinogen in altering the transient behavior of thrombin during clotting. The authors demonstrate that their model is able to capture and potentially explain the dual role of this fibrinogen variant, in which thrombin localization is first enhanced, leading to elevated polymerization, and then effectively quenched via sequestration in a latter phase. This is both interesting and useful contribution to the understanding of this process.
The work appears to have been carried out carefully and is described in detail in the manuscript. On this basis, I only have minor comments for the authors to consider in a revised version of the paper but otherwise recommend for publication in Plos Computational Biology with no reservations. Most of these are very minor in nature and the authors should be able to address them with little impact on the overall structure of the paper.

Specific Comments:

1. The risk vs. protection of the gamma’ chain is not fully resolved in the literature. The authors should cite and discuss some publications that found high gamma chain levels as protective in certain situations.

We thank the reviewer for pointing this out and we have added a new section to the paper in the discussion (this also goes to the comment of reviewer 1):

“In addition to reports that gamma’ fibrinogen increases the risk of thrombosis, a recent study provided evidence that gamma’ fibrinogen was associated with a lower risk of venous thromboembolism, ischemic stroke, cardioembolic stroke, and large artery stroke [1]. It is possible that gamma’ fibrinogen may have different effects depending on the type of vascular disease. A full review of all the various roles for gamma’ fibrinogen in the context of risk vs. protection of thrombosis is beyond the scope of this study, but some good reviews exist in the literature [2,3]. These are very recent studies and the mechanisms underlying roles for risk vs. protection are not understood. Our suggested dual role of gamma’ fibrinogen during polymerization may be able to help elucidate these mechanisms if combined with more spatial-temporal features. Future work could be done with this model to explore venous versus arterial flow conditions as well as injury severity in a spatial-temporal context to explore how these biochemical and biophysical features work together.”

Citing the following work:

[1] Maners, J., Gill, D., Pankratz, N., Laffan, M. A., Wolberg, A. S., de Maat, M. P., ... & MEGASTROKE consortium of the International Stroke Genetics Consortium. (2020). A Mendelian randomization of γ′ and total fibrinogen levels in relation to venous thromboembolism and ischemic stroke. *Blood*, *136*(26), 3062-3069.

[2] Uitte de Willige, S., Standeven, K. F., Philippou, H., & Ariëns, R. A. (2009). The pleiotropic role of the fibrinogen γ′ chain in hemostasis. *Blood, The Journal of the American Society of Hematology*, *114*(19), 3994-4001.

[3] Surma, S., & Banach, M. (2021). Fibrinogen and Atherosclerotic Cardiovascular Diseases—Review of the Literature and Clinical Studies. *International Journal of Molecular Sciences*, *23*(1), 193.

2. The authors have published very advanced models of thrombin generation with time. Were any simulations run where thrombin was initially not present but then generated with time?

This would indeed be an interesting simulation to do. Because all the experimental work that we used to compare to our mathematical results relies on fixed initial concentrations of thrombin, we did not look at thrombin generation.

3. Are the stochastic predictions in agreement with an earlier published estimate of apparent thrombin half-life in a fibrin clot exposed to flow to be about 1.1 hr (Chen, PLoS One, 2019).

We have added the following paragraph to the discussion to address this comment:

"Chen and colleagues recently reported an estimated half-life for thrombin in a fibrin clot exposed to flow to be about 1.1 hrs [1]. To compare our results here to that reported value, we will consider a similar sized fibrin clot of 10$\times$10$\times$15 μm, with thrombin removal at venous shear rates. Thrombin can, at most, interact with a binding junction every 22.5 nm (half the length of fibrin monomers). This means that thrombin has a maximum of 444.45 opportunities to bind along a 10 μm in any direction within the clot. The mean time bound to a wild-type junction from our stochastic model is 60–1000 seconds over the range of removal rates we investigated. Multiplying this time range by the maximal number of binding events gives a total time bound range between 26,667 and 444,450 seconds, or between 7.4 and 123.46 hours. We will now ignoring the time between binding events; the time taken for thrombin to diffuse 10 μm is on the order of 10$^-5$ seconds, using a diffusion coefficient of 1.42 $\times$ 10^{-8} cm^2/s. Next, we assume the volume fraction of fibrin to be about 0.02, using concentrations of 3 mg/ml fibrin from data on the hydraulic permeability of fibrin clots [2] note that this is a higher concentration than that used by Chen and colleagues, which was in a range if 0.088 to 0.551 mg/ml [1]. Thus, with a volume fraction of 0.02, thrombin spends 2\% of the time estimated above, which comes

to a range of 0.15 and 2.47 hours, in the clot before removal. Thus, multiplying this range by $\ln{2}$, assuming first order removal, leads to a range in half-life of 0.1 to 1.71 hours, in agreement with previous estimates of thrombin removal under flow.

We cite the following papers:

[1] Chen J, Diamond SL. Reduced model to predict thrombin and fibrin during thrombosis on collagen/tissue factor under venous flow: Roles of γ’-fibrin and factor XIa. PLoS Computational Biology. 2019;15(8):e1007266.

[2]Wufsus AR, Macera NE, Neeves KB. The Hydraulic Permeability of Blood Clots as a Function of Fibrin and Platelet Density. Biophysical Journal. 2013;104(8):1812–1823. doi:10.1016/j.bpj.2013.02.055.

4. The numerous equations both in the main text and the SI should be numbered so that they can be referenced and easily found by the readers.

This has now been done. We number only referenced equations.

5. Parsing through the modeling section with the various modeling components is rather challenging because it is not clear how the different model sub-components fit together. Can the authors either generate a separate summary figure for this, or use Fig. 2 to also annotate the model sections (numbers would help here as well)?

This was also a comment by reviewer 1. We have added a flowchart to help with the readability of the paper. We have opted to not rewrite to model description to accommodate in text referencing to equations, but we have numbered any already referenced equations.

6. In the polymerization equations in the SI, can the authors verify that all factors related to possible double counting (i.e., i+j versus j+i) have been accounted for? Also, again here, numbering these equations and referring to them directly from the main text will help the interested reader more easily follow the details of the model.

We have now verified all equations.
